# A Deep Learning Framework Based on Denoising and 2D Image Encoding for Arrhythmia Classification

**DOI:** 10.3390/s26165183

**Published:** 2026-08-16

**Authors:** Ji-Yun Seo, Byeong Ho Park, Chang Min Kim

**Affiliations:** AI Education Center, Soonchunhyang University, Asan 31518, Republic of Korea; seoji42@sch.ac.kr (J.-Y.S.); bhpark@sch.ac.kr (B.H.P.)

**Keywords:** ECG, autoencoder, image encoding, denoising, arrhythmia classification, inter-patient evaluation, polarity invariance

## Abstract

Electrocardiogram (ECG) signals are essential for arrhythmia detection; however, they are frequently degraded by noise during acquisition, and their evaluation is vulnerable to data-leakage and patient-overlap issues that can compromise model assessment. Therefore, in this study, we propose an image-encoding-based arrhythmia classifier combined with a morphology-aware denoising autoencoder. We evaluate signals under a corrected, patient-independent protocol in which every model-selection decision was made on a separate validation partition. On a leakage-free test partition, the autoencoder achieved an SNR improvement of 5.63 dB and a correlation coefficient of 0.801, improving R-peak amplitude preservation and redetection accuracy under moderate-to-severe noise while introducing measurable morphology degradation when the input was already lightly contaminated. The proposed model encodes the denoised, beat-centered signal into images through an interleaved-grouping outer product with sorting and flipping, and classifies them with a multi-scale three-dimensional convolutional network. Under this protocol, the proposed model obtained the highest macro-F1 among five image-encoding architectures, but did not outperform four models operating directly on the denoised signal (macro-F1 32.5% versus 37.3–40.0%), indicating that the proposed encoding does not improve overall five-class classification under rigorous inter-patient evaluation. A controlled test showed that the encoding is exactly invariant to global signal-polarity inversion, unlike the sequential models. This targeted invariance, rather than a general accuracy advantage, is the contribution reported here.

## 1. Introduction

Although the average human life expectancy and overall quality of life have improved over time, the number of deaths caused by chronic diseases such as cancer, cardiovascular disorders, and diabetes has continued to rise, largely as a consequence of dietary habits and lifestyle factors. According to the American College of Cardiology, the global prevalence of cardiovascular disease (CVD) nearly doubled over three decades, increasing from 271 million cases in 1990 to 523 million in 2019, while cardiovascular-related deaths rose from 12.1 million to 18.6 million over the same period [1]. CVD encompasses all disorders affecting the heart and blood vessels and, together with stroke, represents one of the leading causes of mortality and disability worldwide. Given this burden, early detection of CVD is critical, and daily monitoring of cardiac health through ECG plays an essential role in early detection and management of cardiovascular diseases [2]. The ECG records the electrical activity generated by cardiac contractions, synchronized with the heartbeat, and therefore contains rich information about the condition of the heart and vascular system, making it one of the most widely used diagnostic tools for cardiac disorders. Among the ECG’s components, the QRS complex is commonly used as a reference for diagnosis and monitoring because of its prominent amplitude and morphology. Both beat morphology and heartbeat-interval features carry discriminative information for arrhythmia classification [3,4].

In practice, however, the measured ECG signal rarely reflects cardiac electrical activity alone; it is commonly contaminated by noise originating from skeletal muscle activity, body movement, respiration, environmental interference, and the electrical characteristics of the measuring device. When such noise is not adequately removed, the quality of the ECG signal deteriorates, which in turn compromises the precision of cardiac analysis and increases the risk of misdiagnosis. To address this, the present study proposes a deep learning-based framework that integrates denoising and image encoding for arrhythmia classification: a fully convolutional autoencoder first suppresses noise while preserving ECG morphology, after which the denoised one-dimensional signal is transformed into a two-dimensional image representation and classified using spatial pattern recognition rather than relying solely on raw temporal signals. In validating this framework under a rigorous, inter-patient evaluation protocol, we further identify and correct several methodological issues present in our original evaluation pipeline—including patient overlap between training and test partitions and inconsistent signal segmentation—and report the resulting, more conservative performance picture, together with a specific, mechanistically confirmed property of the proposed encoding (Section 4.4), rather than a general classification-accuracy improvement. The remainder of this paper is organized as follows. Section 2 reviews related studies on ECG denoising, image-based signal representation, and arrhythmia classification, and identifies the specific gap this work addresses. Section 3 presents the proposed denoising and arrhythmia classification framework. Section 4 describes the experimental setup and performance evaluation results. Finally, Section 5 concludes the paper and discusses future research directions.

## 2. Related Work

ECG records the electrical activity of the heart and plays a pivotal role in the early diagnosis of cardiovascular diseases and the detection of arrhythmias. However, ECG signals are inevitably exposed to various types of noise during measurement, including power-line interference (50–60 Hz), muscle activity, respiration, poor electrode contact, and body movements. Such noise degrades signal quality and reduces the accuracy of analysis and classification [5]. To address this issue, the field of signal processing has long emphasized the need for effective noise reduction along with precise arrhythmia classification. Early studies primarily employed frequency- and time-space domain approaches such as band-pass filters (BPFs), wavelet transform-based filters, median filters, and moving average filters [6,7,8,9,10,11]. While effective in removing noise within specific frequency bands, these methods often cause signal distortion when noise overlaps with the valid ECG frequency range. In addition, they present a trade-off between noise suppression and signal preservation depending on the window size. Artificial intelligence, particularly deep learning, has since been increasingly adopted for ECG denoising. Chiang et al. [12] proposed a fully convolutional denoising autoencoder (FCN-DAE) and demonstrated that it achieves lower reconstruction error and higher signal-to-noise ratio improvement than conventional digital filters and recurrent neural network-based models. For arrhythmia classification, traditional approaches relied on rule-based algorithms based on QRS-complex detection and RR interval analysis. However, these methods were highly susceptible to noise and limited in generalizing across various arrhythmia types. Machine learning techniques such as support vector machines (SVMs), random forests, and k-nearest neighbor (KNN) classifiers were later introduced. Although widely adopted, these methods depend heavily on handcrafted feature extraction and often show performance variability depending on dataset characteristics [13,14,15]. Deep learning models have since become one of the dominant approaches for arrhythmia classification. One-dimensional convolutional neural networks (1D CNNs), long short-term memory (LSTM) networks, gated recurrent units (GRUs), and convolutional recurrent neural networks (CRNNs) have been actively investigated to automatically extract discriminative features directly from raw signals [16,17]. A separate line of work encodes one-dimensional ECG signals into two- or three-dimensional images. It applies advanced image classification models, enabling high-accuracy arrhythmia detection through visual pattern recognition. Ullah et al. [18] transformed 1D ECG signals into 2D images and classified them using a convolutional neural network, demonstrating that the 2D image-based model outperforms its corresponding 1D counterpart on the MIT-BIH arrhythmia database. Zhao et al. [19] incorporated an attention feature fusion module into a 2D image-based EfficientNet architecture and showed that this modification further improves classification accuracy while reducing training cost. These studies demonstrate that image-based representations can capture structural patterns derived from temporal ECG dynamics, unlike conventional approaches that rely solely on handcrafted time-domain features. Similar integrated approaches combining handcrafted and deep features have also been explored for cardiac diagnosis using other physiological signals, such as heart sound recordings [20,21]. Several recent studies have proposed end-to-end frameworks that jointly perform denoising and classification rather than treating them as separate tasks. Peng et al. [22] combined a wavelet-regulated convolutional network with a Transformer encoder within a single framework to extract deep features conducive to both ECG denoising and classification and achieved superior performance on both tasks simultaneously. Although previous studies, including those that jointly address denoising and classification, have improved task-specific or end-to-end performance, most of them process ECG signals primarily in the time or time-frequency domain, and the role of explicit morphology-preserving denoising combined with image-based representation learning remains insufficiently explored. Limited attention has been paid to integrated frameworks that explicitly preserve ECG morphological characteristics during denoising and transform denoised one-dimensional signals into image-based representations for downstream arrhythmia classification. This study therefore examines whether a unified framework that jointly considers waveform fidelity and image-based feature learning improves arrhythmia classification when assessed under a rigorous, patient-independent protocol. We propose an integrated deep learning-based framework that combines morphology-preserving ECG denoising with image-based representation learning, and we evaluate it against both image-encoding and raw-signal architectures. As reported in Section 4, this evaluation does not support a general classification-accuracy advantage for the image-based representation over simpler sequential models; it does, however, identify a specific invariance property of the proposed encoding (Section 4.4). The technical focus of this study is therefore characterizing the exact global-polarity invariance induced by interleaved outer-product encoding, and evaluating its benefits and limitations under a patient-independent protocol.

## 3. Materials and Methods

**Figure 1:**
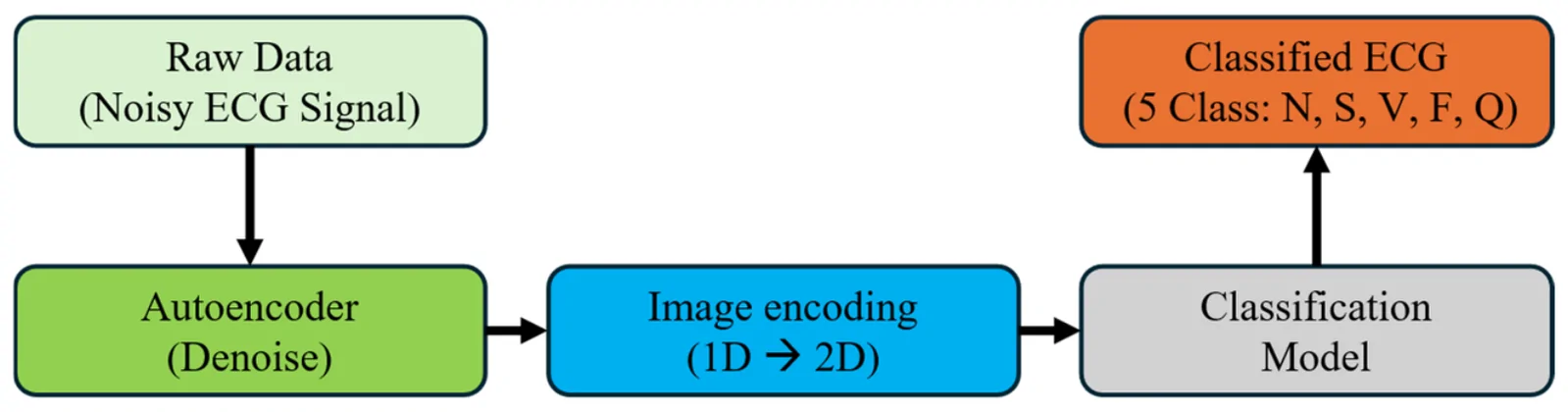
Preprocessing and arrhythmia classification of ECG signals.

Building on the framework outlined above, this section presents the proposed methodology in detail. The proposed framework consists of two main stages: ECG denoising and image encoding. For denoising, an autoencoder model with an encoder–decoder architecture was employed to suppress noise while preserving the morphological characteristics of the ECG, which are essential for accurate arrhythmia classification. The autoencoder was implemented using one-dimensional convolutional layers to extract and reconstruct these characteristics while maintaining the structural integrity of the signal. The denoised one-dimensional ECG signals were then encoded into two-dimensional images by grouping the signal data according to its temporal variations, thereby transforming subtle waveform differences into learnable visual patterns. Because this transformation can introduce geometric distortions, a sorting-and-flipping procedure was subsequently applied to construct a symmetric, spatially expanded representation suitable for model training. Finally, the resulting denoised and image-encoded ECG data were used to train the arrhythmia classification model, enabling the accurate identification of multiple arrhythmia types. An overview of the proposed preprocessing and classification pipeline is illustrated in Figure 1.

The denoising autoencoder was trained using 1024-sample segments, whereas the classification model used 192-sample beat-centered crops extracted from the (denoised) segments. This distinction is maintained throughout the remainder of this section.

### 3.1. Autoencoding for Denoising

**Figure 2:**
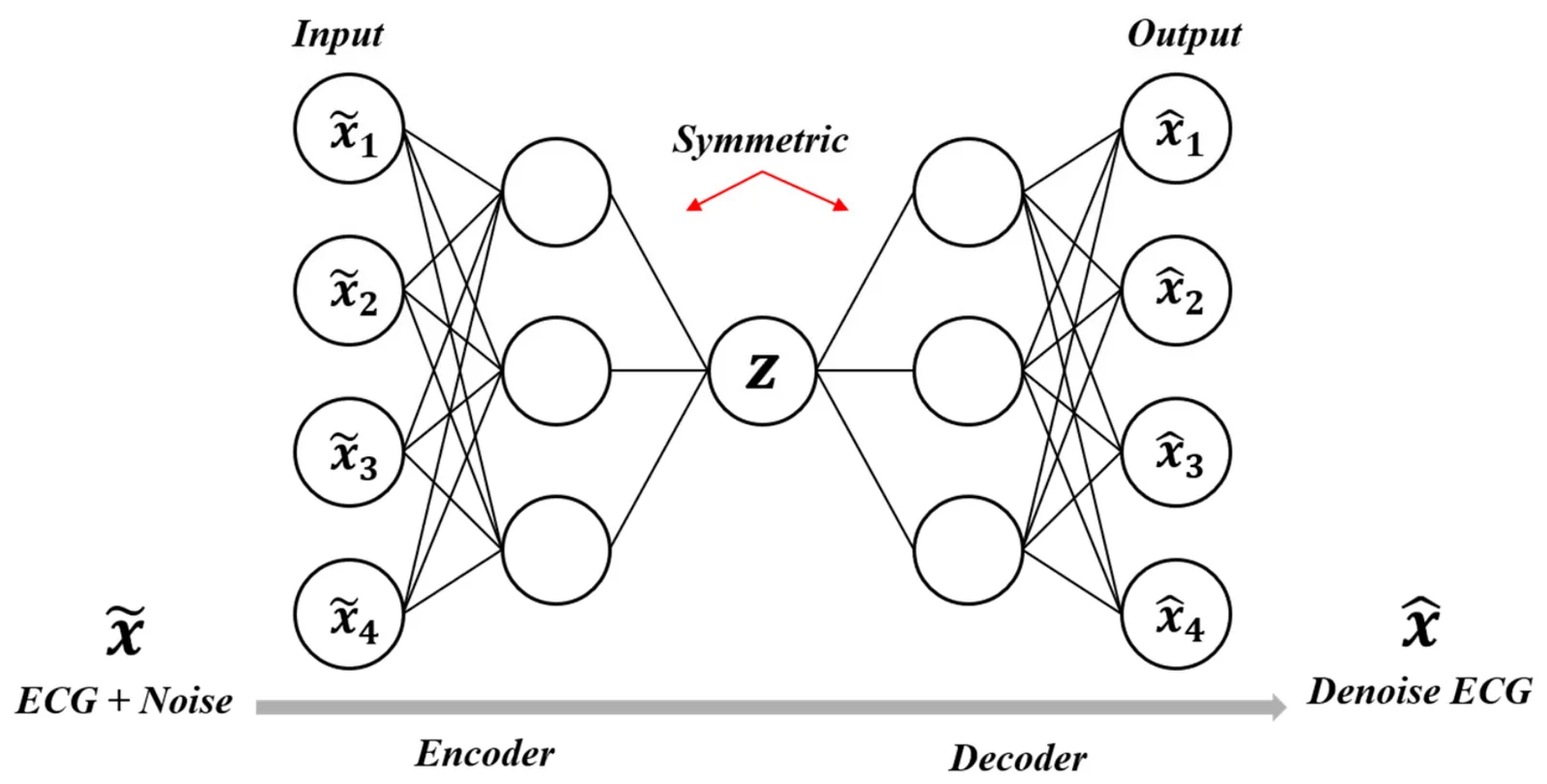
Encoder–decoder structure of the autoencoder used for ECG denoising.

An autoencoder is a neural network composed of an encoder, which progressively reduces the dimensionality of the input data to extract features, and a decoder, which expands the dimensionality to reconstruct the original data. It is commonly applied to tasks such as network pretraining and denoising. Figure 2 illustrates the symmetric encoder–decoder structure adopted in this study, in which the noisy ECG input x is progressively compressed by the encoder into a latent vector z, and the decoder subsequently reconstructs the denoised output $\hat{x}$ from this latent representation. As shown in the figure, the encoder input layer and the decoder output layer share a symmetric structure with the same number of nodes. The noisy ECG input x is passed through the encoder to obtain a compressed latent vector z that represents the essential features of the input, as shown in Equation (1). The decoder then reconstructs the denoised output $\hat{x}$, with the same dimensionality as the original input, from the compressed vector z, as defined in Equation (2). By compressing the input into the latent vector z, the autoencoder learns a compact representation of the essential input features, allowing it to function effectively as a feature extractor while reducing computational memory requirements. In its basic formulation, an autoencoder can be trained by minimizing the mean squared error (MSE) between the reconstructed output $\hat{x}$ and the clean signal x, as defined in Equation (3). This basic reconstruction objective is extended in this study with two additional terms, a first-order temporal-derivative MSE and a center-window MSE, to better preserve beat morphology; the resulting composite loss is specified in Section 3.3. Here, $W_{e}$ and $b_{e}$ denote the weights and biases of the encoder, while Wd and bd denote those of the decoder. When a noisy ECG signal is given as input, if the reconstructed output $\hat{x}$ still contains noise, the loss function value increases; conversely, if $\hat{x}$ closely resembles the clean ECG signal, the loss decreases.

(1) $$z=\sigma \left(W_{e}x+b_{e}\right)$$

(2) $$\hat{x}=\sigma \left(W_{d}z+b_{d}\right)$$

(3) $$\left(\theta ,\theta^{\prime }\right)=\arg\underset{\theta ,\theta^{\prime }}{\min}\frac{1}{N}\sum_{i=1}^{N}|x_{i}-\hat{x_{i}}{|}_{2}^{2}$$

where θ = {$W_{e}$, $b_{e}$} and θ′ = {$W_{d}$, $b_{d}$} denote the trainable parameters of the encoder and decoder, respectively. Equation (3) states the general reconstruction objective common to autoencoder training, and the specific composite loss used to train the autoencoder in this study, which extends this basic MSE objective with additional terms to better preserve beat morphology, is described in Section 3.3. 

### 3.2. Training Data for Denoising

**Figure 3:**
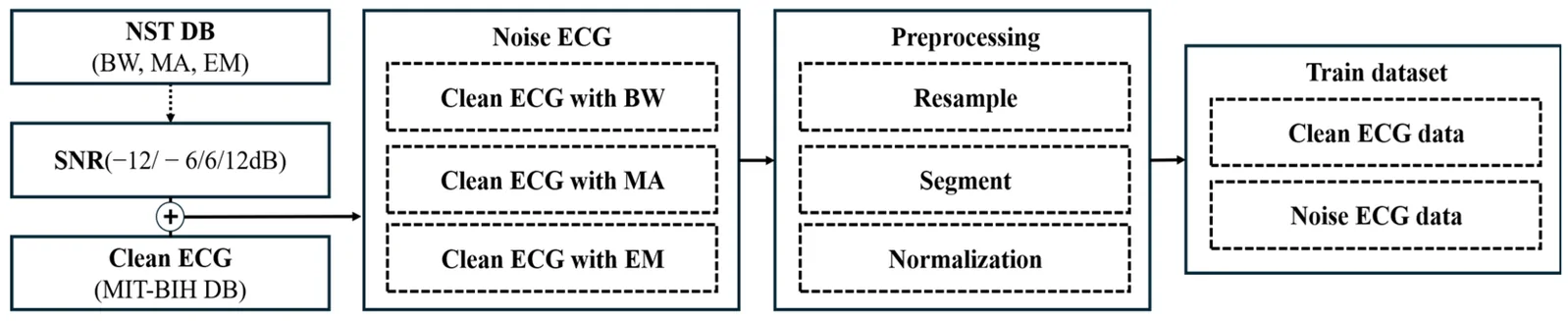
Example of training data generation.

Neural network–based methods learn to extract features and patterns from large datasets. Accordingly, training the proposed autoencoder for denoising requires a substantial amount of training data. For this purpose, the training dataset was constructed using noisy ECG signals as inputs and clean ECG signals as targets. In this study, the MIT-BIH Arrhythmia Database (MIT-BIH DB) [23] was used as the source of clean ECG signals. In contrast, three types of noise signals from the Noise Stress Test Database (NST-DB) [24] were superimposed at four different signal-to-noise ratio (SNR) levels to generate the corresponding noisy ECG signals. An example of the training data generation process is illustrated in Figure 3. The MIT-BIH Arrhythmia Database consists of 48 ECG records obtained from 47 subjects. Each record contains two ECG signals measured over approximately 30 min at a sampling rate of 360 Hz, using two leads selected from MLII, V1, V2, V4, and V5. All records were filtered with a band-pass filter (0.1–100 Hz), and annotations include 15 beat classes along with the corresponding temporal information for each beat. The 15 beat types were regrouped into five categories according to the Association for the Advancement of Medical Instrumentation (AAMI) EC57 standard (Table 1).

All 48 records were considered for dataset construction and partitioned into mutually exclusive training, validation, and test sets at the subject level. Records originating from the same subject were assigned to the same partition to prevent subject-level overlap between training, validation, and test sets. Records were allocated to preserve minority-class representation as much as possible under this patient-independent constraint, since class distributions are highly non-uniform across records (Table 2). Under the fixed patient-independent split used in the reported experiments, the F class was sparsely represented in the test partition, with only 16 beats; per-class recall is therefore reported as a mean ± standard deviation across four random seeds alongside point estimates in Section 4.3. The training, validation, and test sets comprise 33, 7, and 8 records, respectively, containing 76,261, 16,514, and 16,719 beats. Records 201 and 202, which belong to the same subject, were retained together in the training partition, and no record or subject overlapped across the three partitions.

The ECG signals were segmented into 1024-sample windows centered on each annotated R-peak, using the expert beat annotations provided with the MIT-BIH Arrhythmia Database. Annotation sample indices, originally defined at 360 Hz, were mapped to the resampled 250 Hz signal using the same resampling ratio and rounded to the nearest sample index. A single, consistent R-peak-centered segmentation procedure was used throughout both the denoising autoencoder training and the classification pipeline. Boundary beats for which a full 1024-sample window could not be extracted (i.e., beats within 512 samples of the start or end of a record) were excluded. The validation set was defined at the subject level as part of the 48-record partitioning described above (Table 2), rather than by randomly holding out a fraction of training segments, and was used for early stopping and hyperparameter selection. Table 1 summarizes the beat classes redefined according to the AAMI EC57 standard.

Following denoising and prior to image encoding, each 192-sample beat crop was polarity-aligned by inspecting the mean amplitude of its central portion, with segments exhibiting a negative central mean sign-flipped; this step is applied upstream of, and is not part of, the proposed model itself.

Noisy ECG signals for training the fully convolutional autoencoder were generated using the NST-DB, which provides three types of recorded artifacts—baseline wander (BW), electrode-motion artifact (EM), and muscle artifact (MA)—each as a 30-min recording. For each of the three noise types, four SNR levels (–12, –6, 6, and 12 dB) were used, yielding twelve noise conditions in total. Noisy segments were synthesized deterministically: for each (record, beat, noise type, SNR) combination, a noise excerpt was scaled via Equation (4) and added to the corresponding clean segment, with the excerpt starting position and combination seeded so that the same noisy waveform is reproduced across training runs.

**Figure 4:**
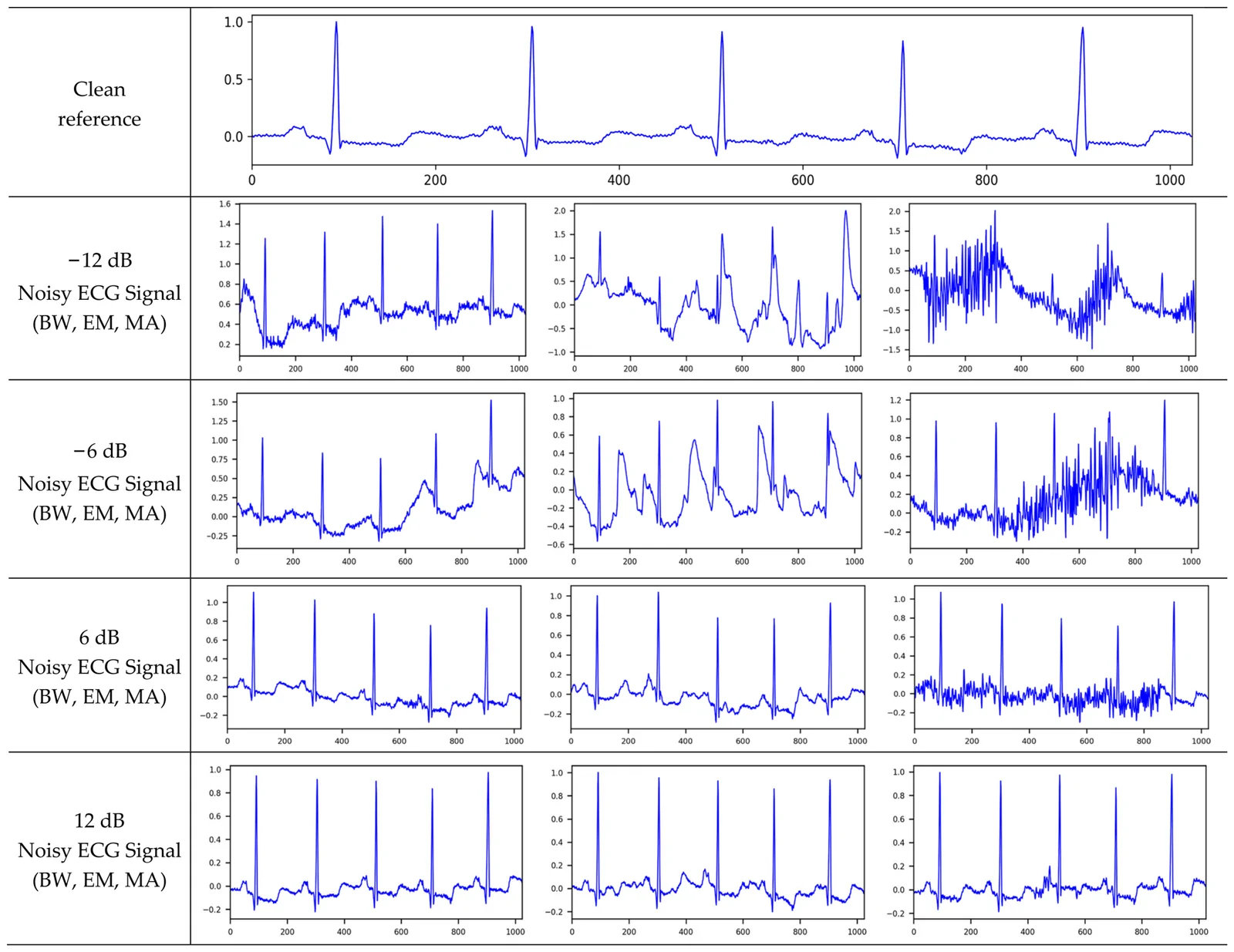
Example of noisy ECG signal generated at different SNR levels.

Both the MIT-BIH DB and NST-DB signals, originally sampled at 360 Hz, were resampled to 250 Hz to reduce computational cost while preserving the ECG frequency components relevant to beat morphology, using anti-aliasing polyphase resampling with an upsampling/downsampling ratio of 25/36. As expressed in Equation (4), noisy ECG signals were synthesized by adjusting the amplitude coefficient a, resulting in SNR levels ranging from −12 dB to 12 dB. Examples of noisy ECG signals generated at different SNR levels are shown in Figure 4.

(4) $$SNR\left(\mathrm{dB}\right)\;=\;20\;\log_{10}\left(\frac{|x_{clean}{|}_{2}}{a|x_{noise}{|}_{2}}\right)$$

### 3.3. Autoencoder Model for ECG Denoising

**Figure 5:**
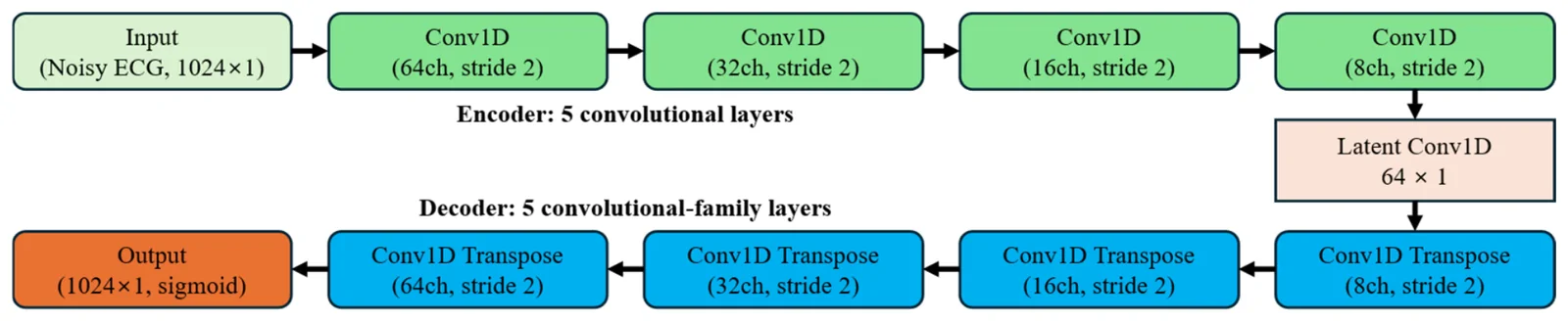
FCN-based denoising autoencoder architecture (10 convolutional-family layers total; input excluded), showing the number of channels and the stride at each layer, with the decoder’s final layer identified as the denoised-ECG output.

A conventional autoencoder typically employs fully connected layers, in which all nodes are interconnected. However, fully connected layers flatten the n-dimensional input data into 1D vectors, leading to the loss of spatial information and limiting the ability to capture global relationships within the data. Moreover, when the input data are high-resolution, the number of parameters increases drastically, resulting in prolonged training times and requiring large datasets to learn effectively from the transformed vectors. In contrast, convolutional neural networks (CNNs), which extract features by applying multiple small convolutional filters across the input data, can preserve spatial and morphological information while achieving strong feature extraction performance even with relatively small datasets. Therefore, in this study, an autoencoder based on a fully convolutional network (FCN) was implemented to achieve effective noise reduction while preserving the morphological characteristics of ECG signals. The designed FCN-based autoencoder consists of 10 convolutional layers in total (5 in the encoder, including the latent-projection layer, and 5 in the decoder, including the final output layer), excluding the input layer. The exponential linear unit (ELU) was adopted as the activation function in the hidden layers, while the sigmoid function was used in the output layer. In the encoder, the first layer receives an input of size 1024 × 1, which is reduced to 512 × 1 through a specified stride and passed to the next layer. This process is repeated, and by the fifth layer, the original 1024 × 1 input is compressed into a 64 × 1 representation. In the decoder, the data are reconstructed in reverse order, expanding the 64 × 1 latent vector back to the original 1024 × 1 signal. The filter size was set to 16, with the number of filters configured as 64, 32, 16, 8, and 1 across the layers. To improve training efficiency and mitigate the vanishing gradient problem, batch normalization was applied to each layer, and early stopping was introduced to prevent overfitting. The autoencoder was trained using the Adam optimizer with a learning rate of 0.001 and a batch size of 128, selected to keep training on the expanded 48-record, twelve-noise-condition dataset computationally tractable. Extending the basic reconstruction objective of Equation (3) introduced in Section 3.1, and to better preserve beat morphology than a plain reconstruction loss would, the autoencoder was actually trained with a composite loss combining (i) reconstruction MSE between the denoised and clean signal, (ii) an MSE term on the first-order temporal derivative of the signal (to encourage preservation of local slope, and hence sharp features such as the QRS complex), and (iii) an additional MSE term computed only over a center-weighted 192-sample window centered on the R-peak (to preferentially preserve the region used by the downstream classifier). The three terms were weighted 1.0, 0.25, and 1.5, respectively. This composite loss replaces a plain MSE objective specifically to reduce the beat-morphology distortion described in Section 4.2. Early stopping was applied with a patience of 10 epochs and a maximum of 1000 training epochs; no weight decay was applied. The architecture of the implemented FCN-based autoencoder is illustrated in Figure 5.

### 3.4. Image Encoding Using 1D Signal Data

ECG data are represented as 1D signals, consisting of values collected at regular intervals over time. Owing to this acquisition method, ECG signals are highly susceptible to noise, which can reduce their accuracy and reliability, and their temporal variability makes interpretation complex. Extracting meaningful features therefore requires specialized knowledge and sophisticated algorithms. By transforming the signal data into images, however, visual patterns can be more easily identified and analyzed. Furthermore, image-based representations allow the application of diverse visualization techniques, enabling intuitive identification of patterns and abnormalities within the data. This section presents an extension and adaptation of the three-axial sensor data image encoding approach proposed by Kim and Lee [25] to single-axis ECG signals. In their study, data collected from gyroscope and accelerometer sensors, each consisting of three axes, were encoded into image representations. However, because the arrhythmia data used in this study consist of single-axis signals, the method in [25] cannot be directly applied. To address this limitation, the temporal variation in signal values, one of the key characteristics of signal data, was leveraged for image encoding.

For classification, each 192-sample beat-centered crop x is decomposed into four phase-offset interleaved subsequences, $G_{j}={\left[x_{j-1+4k}\right]}_{k=0}^{47},\;j\in \{1,2,3,4\}$. Consecutive samples are assigned cyclically, so each $G_{j}$ contains 48 samples drawn at four-sample intervals. These groups represent sampling phases rather than contiguous physiological regions. Outer products are computed for the adjacent pairs (G_1_, G_2_), (G_2_, G_3_), and (G_3_, G_4_).

For the denoising autoencoder, a segment length of 1024 samples at 250 Hz (4.096 s) was retained to provide the network with sufficient temporal context. For classification, however, a 1024-sample window typically spans multiple heartbeats—an average of 4.85 beats per window in our measurements, with essentially all windows centered on a minority-class beat containing at least one neighboring beat of a different class. To ensure the classification task reflects beat-centered morphology rather than multi-beat context, the classifier’s input was defined as a 192-sample (0.768 s) window centered on the same R-peak, cropped from the (denoised) 1024-sample segment; this width keeps mixed-class contamination below 0.1% while retaining local pre- and post-R-peak morphological context.

To refine the resulting image representations, two deterministic transformations were applied. Row-then-column sorting consolidates dispersed outer-product values into ordered spatial regions. Flip-concatenation subsequently mirrors and concatenates each sorted 48 × 48 matrix horizontally and vertically to form a symmetric 96 × 96 representation. This expansion increases spatial redundancy and receptive-field coverage; it does not reconstruct the original temporal coordinates removed by sorting.

**Figure 6:**
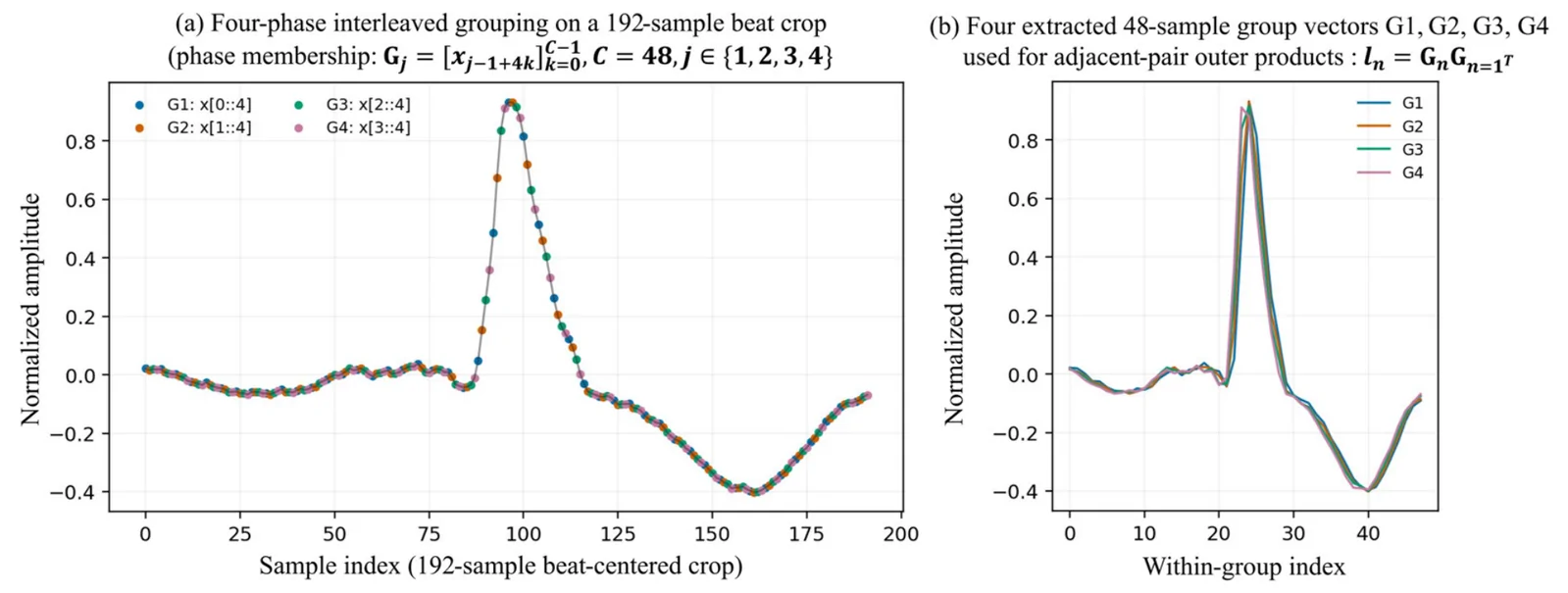
Four-phase interleaved grouping of a representative 192-sample beat crop: (**a**) phase membership on the original sample axis and (**b**) the four extracted 48-sample group vectors.

In Figure 6a, samples at indices 0, 4, 8, … form G_1_; indices 1, 5, 9, … form G_2_; and the same rule defines G_3_ and G_4_. Figure 6b shows the four resulting 48-sample subsequences. All four groups span the complete crop while retaining distinct phase offsets; no anatomical region is assigned to an individual group. The three adjacent pairs (G_1_, G_2_), (G_2_, G_3_), and (G_3_, G_4_) form I_1_, I_2_, and I_3_ in Equation (5).

(5) $$G=\left\{G_{1},G_{2},G_{3},G_{4}\right\},\;I_{n}=G_{nG_{n+1}^{T}},\;n\in \{1,2,3\},\;I=\left\{I_{1},I_{2},I_{3}\right\}$$

**Figure 7:**
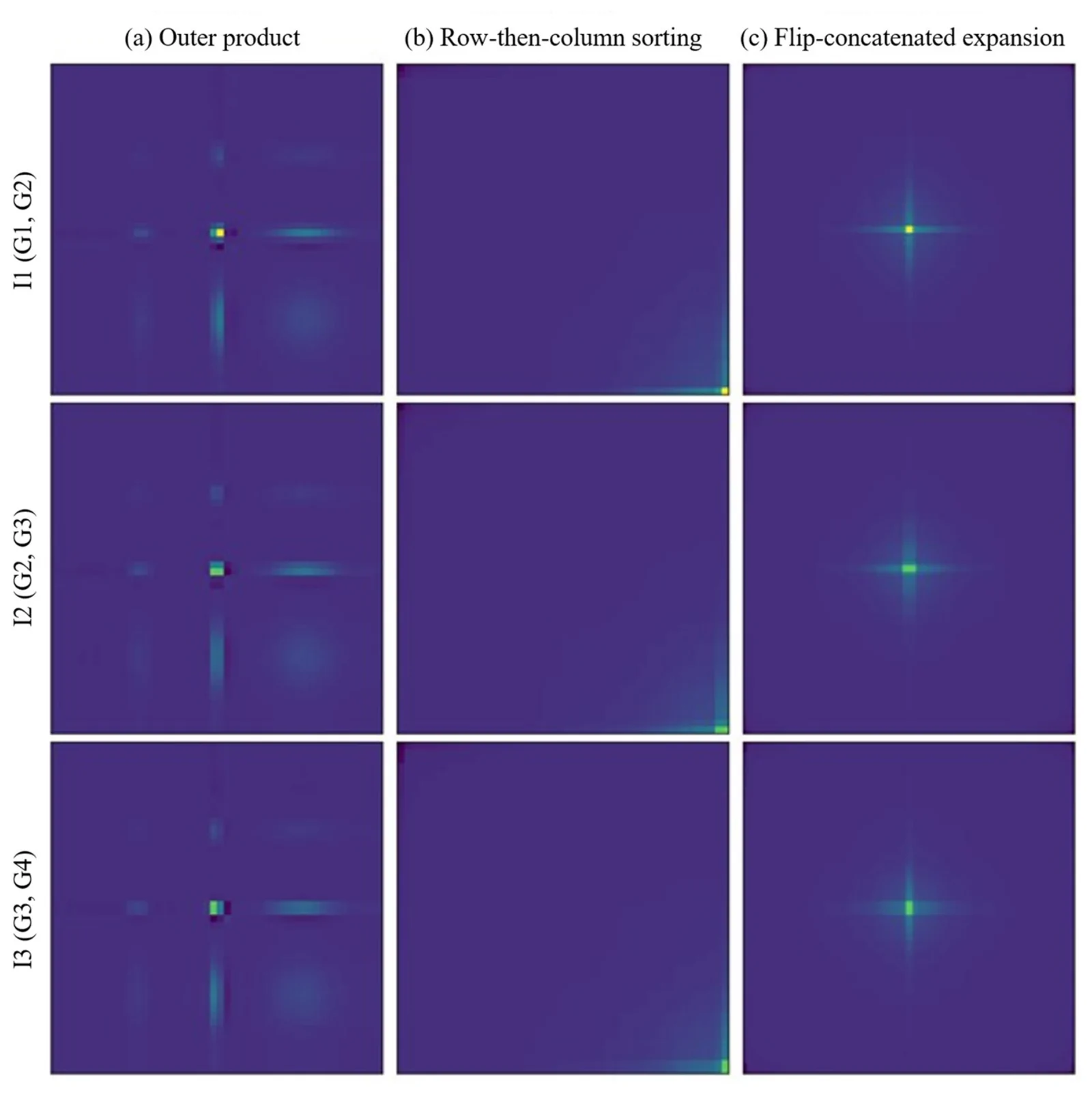
Image-encoding pipeline for the three adjacent group pairs: (**a**) outer-product frames, (**b**) row-then-column sorted frames, and (**c**) flip-concatenated symmetric expansions.

Figure 7a shows that the three adjacent-pair outer products produce waveform-dependent spatial patterns. The resulting C × C matrices (C = 48) can be processed as images, but their values are spatially dispersed. Figure 7b shows the row-then-column sorting in Equation (6), which preserves the multiset of intensities while discarding the original row and column coordinates. Figure 7c shows the deterministic 96 × 96 symmetric expansion in Equation (7).

(6) $$I_{n}^{sorted}=sort\left(sort\left(I_{n},\;axis=1\right),\;axis=0\right),n\in 1,2,3$$

In Equation (6), sort() rearranges I_n_ first within each row (axis = 1) and then within each column (axis = 0), yielding the sorted frames shown in Figure 7b.

The sorting operation aggregates dispersed values but deliberately removes their original coordinates. Equation (7) then constructs the symmetric spatial expansion shown in Figure 7c; it does not recover the temporal coordinates removed by sorting.

(7) $$\begin{array}{c} H_{n}=[I_{n}^{sorted}|fliplr(I_{n}^{sorted})]],\;R_{n}=[\begin{array}{c} H_{n}\\ flipud\left(H_{n}\right) \end{array}],\;n\in \{1,2,3\},\\ R=\{R_{1},R_{2},R_{3}\} \end{array}$$

In Equation (7), fliplr() and flipud() denote horizontal and vertical reflection. $H_{n}$ concatenates $I_{n}^{sorted}$ with its horizontal reflection, and $R_{n}$ concatenates $H_{n}$ with its vertical reflection. The result is a symmetric 2C × 2C frame (96 × 96 for C = 48). This operation deterministically expands the sorted image but does not restore the original temporal coordinate system.

### 3.5. ECG Arrhythmia Classification Model

**Figure 8:**
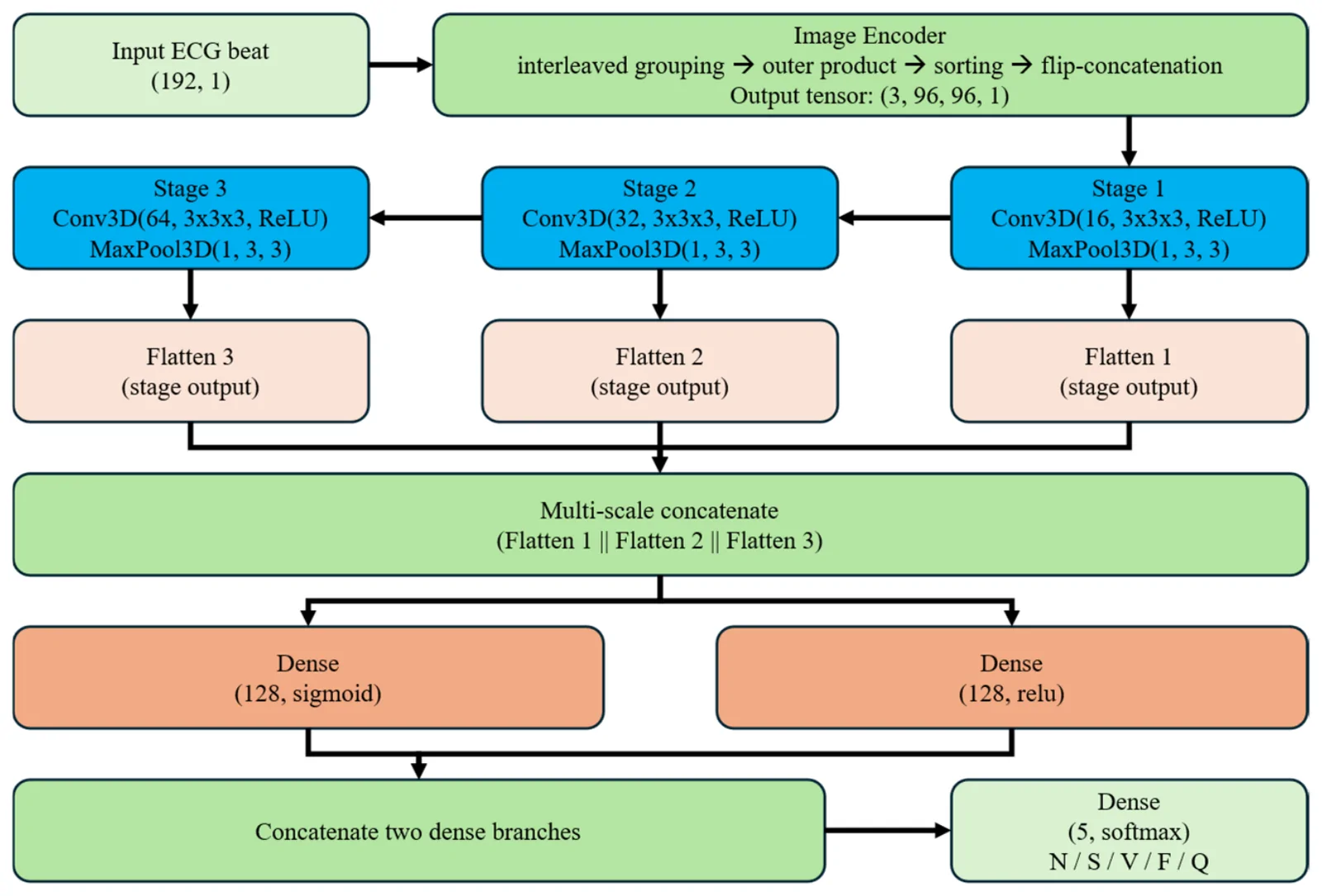
Architecture of the proposed model: interleaved image encoding followed by a sequential three-stage, multi-scale, dual-branch 3D CNN.

In this study, the images generated through the outer-product operation, followed by the first and second correction steps, were used for training, adopting an adapted version of the learning model proposed in [25]. In [25], a model architecture was introduced that simultaneously learns from 1D signal data, 2D images, and 3D images generated through image encoding. In this study, however, arrhythmia classification was performed using only the 3D hierarchical structure of the generated images, without incorporating the corresponding 1D and 2D representations. Figure 8 illustrates the architecture of the proposed arrhythmia classification model, which employs 3D convolutional layers. As shown in the figure, the model input consists of 3D images expanded from the 1D signal data through the proposed image encoding method (Section 3.4). The upper part of the model consists of three stacked 3D convolutional layers with filter counts of 16, 32, and 64, each employing a kernel size of (3, 3, 3) with the ReLU activation function. Following each convolutional layer, a max-pooling layer of size (1, 3, 3) is applied to reduce dimensionality, retain important features, and suppress noise.

Unlike a conventional single-path CNN, the feature map produced after each of the three convolutional/max-pooling stages is separately flattened, yielding three feature vectors at different spatial scales; these three vectors are concatenated into a single multi-scale feature vector. This multi-scale vector is then processed by two parallel dense branches operating on the same input: one branch with 128 neurons using the sigmoid activation function, and a second, independent branch with 128 neurons using the ReLU activation function. The outputs of these two branches are concatenated and passed to a final dense layer of five neurons with the softmax activation function to produce the class probabilities. Combining sigmoid- and ReLU-activated branches in this way allows the model to capture complementary response patterns from the same multi-scale feature representation. With this configuration, the proposed model has 15,554,085 trainable parameters.

For comparison, the proposed model was benchmarked against eight additional models under identical data, preprocessing, and evaluation conditions. Four of these share the same image-encoded input as the proposed model: a simpler three-layer 3D CNN baseline (single final-stage flatten, without the multi-scale concatenation or dual dense branches described above), and 3D adaptations of ResNet-18, GoogLeNet (Inception-v1), and DenseNet-121, in which the 2D convolutional layers of each architecture were replaced with 3D convolutional layers to accommodate the (3, 96, 96, 1) encoded input while preserving each architecture’s characteristic connectivity (residual connections for ResNet-18, inception modules for GoogLeNet, and dense connectivity for DenseNet-121). The remaining four baselines operate directly on the denoised, polarity-aligned, 192-sample signal without image encoding: a 1D CNN, a long short-term memory (LSTM) network, a gated recurrent unit (GRU) network, and a convolutional recurrent neural network (CRNN).

All classification models were trained for up to 100 epochs using the Adam optimizer with categorical cross-entropy loss, and early stopping (patience 5) based on record-level macro-F1 computed on the validation set. To address class imbalance, we evaluated four policies—no adjustment, random oversampling, oversampling combined with mild duplicate augmentation (time-jitter, amplitude/baseline perturbation), and inverse-frequency class weighting—using validation-set record-level macro-F1 prior to any test-set evaluation. The no-adjustment policy achieved the highest validation performance (29.45 ± 1.43%, versus 29.33 ± 0.54% for oversampling, 28.01 ± 0.75% for oversampling with augmentation, and 27.43 ± 1.80% for class weighting). It was therefore frozen as the final policy applied uniformly across all nine compared architectures and reported in Section 4.3. All nine models were trained and evaluated across four random seeds (42–45).

### 3.6. Statistical Analysis

For the revised inferential analysis, record-level five-class macro-F1 aggregated across the twelve noise-type × SNR test conditions was designated as the primary endpoint. For each record, class-wise F1 scores were computed over the fixed five-class label set (N, S, V, F, and Q). Classes absent from the ground-truth labels of a record were retained in the macro average, with undefined zero-denominator quantities assigned a value of zero. The five class-wise F1 scores were then averaged, and the resulting record-level macro-F1 values were averaged with equal weight across records. Clean-condition record-level macro-F1 was reported separately as a descriptive secondary summary and was not the endpoint tested. Mean differences and 95% confidence intervals were estimated using a 10,000-replicate hierarchical paired bootstrap that resampled test records as the outer patient-level clusters while retaining seeds and all 12 noise-type × SNR conditions within each selected record. For hypothesis testing, paired differences were first averaged across the four seeds and 12 conditions within each record. One sign was then assigned to each of the eight test records, so all repeated observations from a selected record were exchanged together. Two-sided exact paired-permutation *p*-values enumerated all 2^8^ = 256 record-level sign assignments. Holm correction was applied across the eight proposed-model-versus-comparator tests within each endpoint family. Balanced accuracy and macro-specificity were secondary endpoints. We note an inherent power constraint of this design. With eight test records, the smallest attainable two-sided exact permutation *p*-value is 2/2^8^ = 0.0078, which becomes 0.0625 after Holm correction across eight comparators. No comparison in either endpoint family can therefore reach the conventional 0.05 threshold, irrespective of the size of the underlying effect. The record-level tests are accordingly reported as a conservative check on the direction and magnitude of differences rather than as a confirmatory test of significance, and our conclusions rest on effect sizes and confidence intervals rather than on *p*-value thresholds.

## 4. Experiments and Results

### 4.1. Evaluation Metrics for Denoising Performance

**Figure 9:**
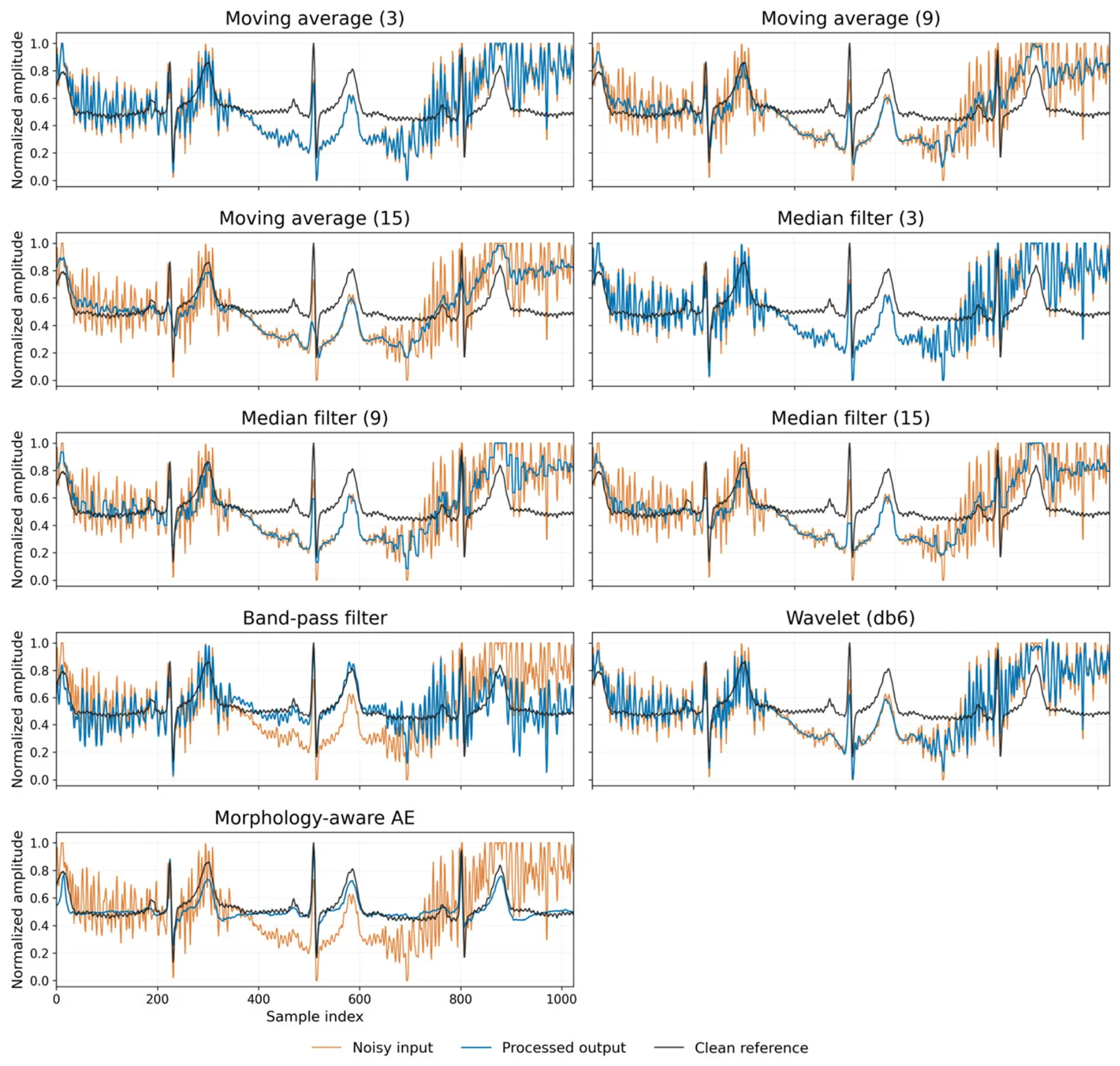
Representative qualitative denoising outputs for a fixed patient-independent test segment (record 117, muscle-artifact noise, −6 dB). Moving-average and median filters used window sizes of 3, 9, and 15 samples; the band-pass output was obtained using a fourth-order Butterworth filter at 0.5–40 Hz, and wavelet denoising used db6 with universal soft thresholding. All methods received the same noisy input. Orange, blue, and black lines denote the noisy input, processed output, and clean reference, respectively. This illustration is qualitative and is not used to claim quantitative superiority.

To evaluate the denoising performance of the morphology-aware autoencoder, we used the leakage-free validation and test partitions described in Section 3.2, spanning three simulated noise types (BW, EM, and MA) and four SNR levels (−12, −6, 6, and 12 dB). The denoised signals were compared with their corresponding clean ECG signals using SNR improvement, RMSE, PRD, and the Pearson correlation coefficient. In the implementation used to generate the reported results, both the clean reference and the reconstruction residual were mean-centered before their root-mean-square amplitudes were calculated. Equations (8)–(10) state this implementation exactly. Mean-centering was applied here because ECG signals commonly contain baseline-wander components that would otherwise distort the RMS-based SNR calculation, whereas Equation (4) uses the raw, non-mean-centered norm. After all, it scales an already-fixed noise waveform to set the synthesis-time target level, a distinct purpose from the post-hoc evaluation performed in Equations (8)–(10). A quantitative, like-for-like comparison against conventional filters is not claimed here because auditable retained outputs for that comparison were not available; Figure 9, which illustrates conventional filtering methods and the proposed autoencoder, is therefore qualitative only.

(8) $${rms}_{c}\left(z\right)=\sqrt{\frac{1}{n}\sum_{i=1}^{n}{\left(z_{i}-\bar{z}\right)}^{2}}$$

(9) $$SNR\left(x,\hat{x}\right)\;=\;20\;\log_{10}\left(\frac{{rms}_{c}\left(x\right)}{{rms}_{c}\left(\hat{x}-x\right)}\right)$$

(10) $${SNR}_{imp}=SNR\left(x,{\hat{x}}_{denoised}\right)-SNR\left(x,x_{noisy}\right)$$

The root mean square error (RMSE) is an evaluation metric that measures the difference between the predicted and actual values, calculated by taking the square root of the mean square error (MSE), as shown in Equation (11).

(11) $$RMSE=\sqrt{\frac{1}{n}\sum_{i=1}^{n}{\left(x_{i}-{\hat{x}}_{i}\right)}^{2}}$$

The percent root mean square difference (PRD), defined in Equation (12), is a metric used to measure the distortion between the original signal and the reconstructed signal.

(12) $$PRD\left(\%\right)=100\sqrt{\frac{\sum_{i=1}^{n}{\left(x_{i}-{\hat{x}}_{i}\right)}^{2}}{\sum_{i=1}^{n}x_{i}^{2}}}$$

The correlation coefficient was calculated using the Pearson correlation coefficient, as shown in Equation (13).

(13) $$\rho \left(x,y\right)=\frac{\sum_{i=1}^{n}\left(x_{i}-\bar{x}\right)\left(y_{i}-\bar{y}\right)}{\sqrt{\sum_{i=1}^{n}{\left(x_{i}-\bar{x}\right)}^{2}}\sqrt{\sum_{i=1}^{n}{\left(y_{i}-\bar{y}\right)}^{2}}}$$

Denoising performance (SNR improvement, RMSE, PRD, and correlation coefficient) was computed separately for each of the three noise types (BW, EM, MA) and four SNR levels (−12, −6, 6, and 12 dB). Table 3 provides the overall validation and test summaries for the final morphology-aware autoencoder, and the strongest severe-noise and low-noise patterns are reported explicitly in Section 4.2. Record-level aggregation was used so that a small number of long recordings could not dominate the reported averages.

Because global signal-fidelity metrics such as SNR and correlation do not guarantee that the specific morphological features relevant to arrhythmia classification are preserved, denoising performance was additionally evaluated in terms of morphology preservation: R-peak amplitude error, R-peak redetection sensitivity and positive predictive value (PPV), RR-interval error, and, as secondary indicators, a slope-threshold-based estimate of QRS-width error and P-wave/T-wave correlation. R-peak amplitude error and redetection sensitivity/PPV are reported as the primary morphology indicators, since QRS-width and P-wave-correlation estimates rely on a heuristic onset/offset detector rather than a validated clinical algorithm and showed direction-inconsistent behavior across partitions in our evaluation (Section 4.2).

### 4.2. Results of Denoising Evaluation

The final morphology-aware autoencoder achieved an overall SNR improvement of 6.71 dB on the validation partition and 5.63 dB on the test partition, with an RMSE of 0.205 (validation)/0.143 (test), a PRD of 40.24% (validation)/36.89% (test), and a correlation coefficient of 0.870 (validation)/0.801 (test) across all three noise types and four SNR levels (Table 3). Improvement was strongest under severe noise: on the test partition, SNR improvement at −12 dB was +15.65 dB for baseline wander, +12.83 dB for electrode-motion artifact, and +13.69 dB for muscle artifact. At 12 dB, however, SNR improvement was negative (approximately −3.93 to −4.09 dB across noise types), indicating that the autoencoder altered signals that required little correction.

Morphology-preservation analysis showed that the proposed autoencoder improved R-peak amplitude preservation and redetection robustness under moderate-to-severe noise, while causing measurable degradation in several morphology-preservation metrics under low-noise conditions (Table 4). On the test set (Table 4a), the absolute R-peak amplitude error decreased from 0.720 to 0.390, redetection sensitivity increased from 0.812 to 0.834, and PPV increased from 0.372 to 0.547 after denoising. These gains were most pronounced at −12 dB and −6 dB—for instance, at −12 dB (Table 4b), amplitude error decreased from 1.35–1.78 to 0.43–0.59 and PPV increased from 0.22–0.42 to 0.43–0.55 across the three noise types—whereas at 12 dB the denoised signals exhibited increased R-peak amplitude error (0.11–0.13 to 0.31–0.32 across noise types) and reduced P-wave correlation (0.89–0.99 to 0.62–0.65), indicating that autoencoder-based denoising may be mildly counterproductive when the input signal is already only lightly contaminated. Because the QRS-width and P-wave-correlation estimates rely on a heuristic onset/offset detector and showed direction-inconsistent behavior between the validation and test partitions (e.g., QRS-width error improved on validation but worsened on test), we treat R-peak amplitude error and redetection sensitivity/PPV as the primary evidence for this pattern, consistent with the negative SNR improvement observed at 12 dB above.

### 4.3. Performance of the Image Encoding-Based Arrhythmia Classification Model

Classification performance is reported both as a pooled test-set summary and broken down by record, given that segments drawn from the same recording are not statistically independent. Because certain minority classes—most notably F, with only 16 test-set beats concentrated in a small number of records—have limited test-set support, per-class recall is additionally reported as a mean ± standard deviation across four random seeds rather than as a single point estimate. These standard deviations characterize training-related variability across seeds; they are not confidence intervals and do not represent sampling uncertainty with respect to the test population.

To assess whether the proposed model offers an advantage over alternative architectures under identical, leakage-free, patient-independent conditions, the class-imbalance handling policy (no oversampling, no class weighting; Section 3.5) was frozen using validation-only performance prior to any test-set evaluation. Under this frozen protocol, the proposed model was compared against all eight baselines described in Section 3.5, with all nine models trained and evaluated across four random seeds (Table 5).

Two patterns emerge from Table 5. First, among the five image-encoding-based models, the proposed model achieved the highest macro-F1 and record-level macro-F1. Second, under the record-level exact paired-permutation procedure specified in Section 3.6, none of the eight primary-endpoint comparisons was significant after Holm correction (Table 6). The proposed-model-versus-1D-CNN difference was −2.005 percentage points (95% CI −5.364 to 1.292; Holm-corrected *p* = 1.000). We therefore do not claim a statistically supported aggregate advantage for either representation tier.

None of the eight comparisons reached significance after Holm correction on this primary metric.

In Table 7, Class F was recognized at 0% recall by every model evaluated except CRNN (1.56%), consistent with its extremely limited training (423 beats) and test (16 beats) support; this class-level failure was therefore not resolved by any of the nine architectures compared, including the proposed model. Relative to the simple 3D CNN baseline, the proposed model’s multi-scale, dual-branch classifier improved V-class recall by 21.9 percentage points (33.86% to 55.80%) and Q-class recall by 16.7 percentage points (4.11% to 20.81%); however, both classes remained highly seed-dependent for the proposed model (Q-class recall ranged from 3.9% to 34.7% across the four seeds), and this variability should be kept in mind when interpreting the mean improvement.

To complement recall, Table 8 highlights the proposed model’s N-class specificity because this class drives most of its macro-specificity gap, while also giving the typical range for the other four classes. Complete class-wise specificity values, raw and row-normalized confusion matrices, and per-class precision, recall, and F1 for all nine models across every BW/EM/MA × SNR condition are provided in the supplementary CSV files (see Data Availability).

Applying the same record-level exact paired permutation to the secondary endpoints yielded no Holm-corrected significant comparisons. Directionally, the proposed model’s balanced accuracy was lower than GRU by 2.836 percentage points (raw *p* = 0.0156, Holm *p* = 0.125) and CRNN by 2.691 points (raw *p* = 0.0234, Holm *p* = 0.1641); macro-specificity was lower than GRU by 2.335 points and CRNN by 2.363 points (raw *p* = 0.0156 and Holm *p* = 0.125 for both). These secondary results are treated as exploratory directional patterns, not confirmatory differences. As noted in Section 3.6, the eight-record design cannot attain Holm-corrected significance under any outcome, so the absence of significant comparisons must not be read as evidence that the models are equivalent. Class-wise specificity nevertheless identifies N-class specificity as the proposed model’s principal weakness, whereas S/V/F/Q specificity remained between 94.6% and 99.9% across all four SNR levels.

Across BW, EM, and MA, the raw-signal tier retained the highest absolute macro-F1, while the proposed model was the best-performing image-encoding model for every noise type (Table 9). EM was generally the most difficult noise type across models. Table 10 complements this analysis by pooling noise types and stratifying performance by SNR.

At every SNR level, the raw-signal model tier (1D CNN, LSTM, GRU, CRNN) outperformed the image-encoding tier in macro-F1, and the proposed model remained the best-performing image-encoding-based model throughout, consistent with the clean-condition pattern (Table 5). Within the raw-signal tier, however, the apparent ranking among the four models was not statistically robust at any SNR level: for example, the gap between 1D CNN (highest mean at −12, −6, and 6 dB) and its closest competitor was only 0.21–0.25 times the combined standard deviation at −12 dB, and CRNN and 1D CNN were effectively tied at 12 dB (39.66 ± 3.36 vs. 39.64 ± 4.90, a difference of 0.00 combined standard deviations). This is consistent with the pattern noted in Section 4.3: pairwise differences among the four raw-signal models were small relative to their seed-to-seed variability, so we describe them descriptively as a single tier across noise conditions as well as in the clean condition, without claiming a formally tested absence of difference (the formal statistical tests in this study, Table 6, compare the proposed model against each comparator individually and do not include the six pairwise comparisons among the four raw-signal models themselves). The proposed model’s own macro-F1 improved consistently from −12 dB to 12 dB (25.72 to 32.04, a difference of about 1.7 times the combined standard deviation), mirroring the overall SNR-dependent trend seen across all nine models. Electrode-motion (EM) noise was, on average across models, the most difficult of the three noise types, consistent with the pattern reported for the denoising autoencoder itself (Section 4.2).

The full sorting-plus-flipping procedure did not demonstrate a consistent benefit across the validation, record-level test, and pooled test endpoints: it achieved the highest pooled test macro-F1, but the no-correction variant achieved the highest validation record-level macro-F1, and the sorting-only variant achieved the highest record-level macro-F1 under both the clean and the all-noise test conditions (Table 11). Because this comparison is based on a single training seed per variant, we do not consider the ranking among these three variants firmly established and flag it as a candidate for follow-up seed repetition. We additionally note a confound in this ablation: the flipping step doubles each spatial axis (48 × 48 to 96 × 96), so the “+ Sorting + Flipping” variant differs from “+ Sorting” alone in both the presence of symmetric spatial expansion and the resulting spatial resolution/redundancy of the input. The comparison should therefore be interpreted as reflecting the combined effect of symmetric spatial expansion and resolution expansion, rather than isolating the contribution of flipping per se; a resolution-matched control (e.g., upsampling the unflipped variant to the same input size) would be needed to separate these two factors.

The proposed model’s multi-scale, dual-branch classifier head uses approximately 20 times more parameters than the 1D CNN, predominantly due to the dense layers operating on the concatenated multi-scale flattened features; this did not translate into a measurable inference-time advantage or disadvantage, with the two models exhibiting comparable per-sample latency (Table 12). We therefore do not claim an efficiency advantage for the proposed model based on the present results; if anything, the proposed model achieves lower aggregate classification performance (Table 5) with substantially more parameters than the 1D CNN baseline.

### 4.4. Mechanistic Analysis: Polarity Invariance of the Outer-Product Encoding

To understand the specific conditions under which the proposed model and the other image-encoding-based models outperform the raw-signal models at the level of individual test records—despite their lower aggregate performance (Table 5)—we examined record-level results in detail. Record 117 (1534 N beats, 1 S beat, effectively an N-dominant record) illustrates this pattern clearly. All five image-encoding-based models retained N-class recall above 95% (95.9–99.4%), compared with 53.4% for the 1D CNN, 84.3% for LSTM, 67.1% for GRU, and 69.3% for CRNN. The proposed model itself achieved 98.71 ± 0.97% N-class recall, consistent across all four seeds (97.3–99.3%). The comparison was most robust for the proposed model against the 1D CNN and CRNN (effect sizes of approximately 11.2 and 4.6 times the combined standard deviation, respectively); against LSTM specifically, the proposed model’s mean advantage was similar in size to LSTM’s own seed-to-seed variability (14.11 percentage points), because LSTM’s individual-seed performance on this record ranged widely (63.95–94.72%), occasionally approaching the image-encoding tier. The weakest image-encoding model on this record, DenseNet-121 (95.80 ± 3.68%), showed a similarly modest effect size relative to LSTM. The proposed model’s own seed-to-seed standard deviation (0.97 percentage points) was nonetheless the smallest of all nine models on this record, indicating that its record-117 robustness is not only high on average but unusually consistent across independent training runs—in contrast to LSTM and GRU, whose performance on this specific record varied enormously depending on the random seed.

A detailed inspection of record 117 revealed an abrupt change in central-waveform polarity approximately 718–729 s into the recording (correlation between early- and late-segment average waveforms of −0.52 to −0.69 after the polarity-alignment step described in Section 3.2, compared with +0.74 to +0.77 before alignment), indicating that the beat-wise polarity-alignment heuristic, while beneficial on average, produced inconsistent sign decisions within this particular record. Errors for the raw-signal models were concentrated almost entirely in the affected segment; for the 1D CNN, mean accuracy was 11.8% before the shift versus 80.4% after, consistent across all four seeds (74.6% of all 1D CNN errors on this record fell within the affected segment, which comprises only 39.4% of the record’s beats), with the great majority of errors being N beats misclassified as V.

This pattern motivated a controlled test of the outer-product encoding’s sensitivity to global polarity inversion. For a global sign reversal of the input signal, the outer product of two temporal groups satisfies (−G_n_)(−G_n+1_)ᵀ = G_n_G_n+1_ᵀ, implying that the encoding is mathematically invariant to a simultaneous sign flip of the entire signal, a property preserved under the subsequent sorting and flipping steps. We verified this directly using both synthetic ECG-like signals and 16 real beats drawn from the dataset: the encoded representation was numerically identical (element-wise, to floating-point precision; maximum absolute difference of 0.0) for a signal and its sign-reversed counterpart, and the trained proposed model produced identical predicted probabilities for both (0.0% change in predicted label). In contrast, applying the same sign reversal to real beats and to a class-balanced validation subset changed the 1D CNN’s predicted class for 79.0–93.1% and 43.0–54.6% of samples, respectively, across four random seeds.

This confirmed architectural property—exact invariance to global polarity inversion for the image-encoding pipeline, versus substantial polarity sensitivity for the raw-signal models—provides a mechanistic explanation for the image-encoding models’ markedly higher and more consistent performance on record 117, the single test record in which such an internal sign inconsistency was observed. We emphasize an important caveat regarding this interpretation: the early/late correlation reversal observed in record 117 as described above occurred specifically after the beat-wise polarity-alignment heuristic was applied (+0.74 to +0.77 before alignment, −0.52 to −0.69 after), indicating that this particular “polarity shift” may reflect an inconsistency introduced by the alignment heuristic itself—which made different sign choices for beats before and after a certain point in the recording—rather than a polarity change intrinsic to the underlying physiological signal. Consequently, the demonstrated robustness of the proposed model on this record is most precisely characterized as robustness to inconsistent sign decisions introduced by upstream preprocessing, rather than to a naturally occurring polarity reversal in the ECG signal itself; whether the same robustness generalizes to genuine, physiologically driven polarity changes (e.g., differing lead placements or cardiac axis shifts) has not been separately established and would require dedicated evaluation, for instance with polarity alignment disabled. We note, however, that record 117’s internal change involves some degree of amplitude and morphology variation in addition to a pure sign reversal, so while the polarity-invariance mechanism is directly confirmed at the level of the encoding and the trained model, its role as the complete explanation for this specific record’s behavior should be interpreted as strongly supported rather than exhaustively isolated. This observation raises the hypothesis that removing sign information may contribute to reduced discrimination of classes (S, F) whose morphology depends partly on waveform direction rather than magnitude alone. This possibility was not isolated experimentally in the present study; we return to the trade-off in Section 5.

## 5. Conclusions

In this study, we set out to develop an integrated deep learning framework combining ECG denoising and image-encoding-based arrhythmia classification. In the course of rigorously validating this framework under an inter-patient, leakage-free evaluation protocol, we identified and corrected several methodological issues affecting the original evaluation—including same-patient record overlap between training and test partitions, inconsistent segmentation, and evaluation-set contamination during model selection—and, having corrected them, obtained a substantially different and more conservative picture of the framework’s classification performance than initially reported.

The proposed autoencoder, evaluated on a leakage-free, patient-independent test partition of 200,628 signal-condition pairs across three noise types and four SNR levels, achieved an SNR improvement of 5.63 dB, an RMSE of 0.143, a PRD of 36.89%, and a correlation coefficient of 0.801. Morphology-preservation analysis further showed improved R-peak amplitude preservation and redetection accuracy under moderate-to-severe noise (e.g., amplitude error reduced from approximately 1.3–1.8 to 0.4–0.6 under −12 dB conditions), alongside a measurable increase in amplitude error and reduced P-wave correlation under already low-noise (12 dB) input. We do not claim quantitative superiority over conventional filters because a complete, auditable, like-for-like comparison was not retained for this revision.

For classification, under a protocol in which the class-imbalance handling policy was frozen using validation-only performance prior to any test-set inspection, and results were averaged over four random seeds, the proposed model (the proposed interleaved image encoding combined with a multi-scale, dual-branch 3D CNN, 15,554,085 parameters) achieved the highest macro-F1 and record-level macro-F1 among five image-encoding-based architectures (a simple 3D CNN baseline, ResNet-18, GoogLeNet, and DenseNet-121, all sharing the identical encoded input). However, the proposed model did not outperform any of four raw-signal models (1D CNN, LSTM, GRU, CRNN) operating on the same denoised, beat-centered signal: the proposed model achieved a test-set macro-F1 of 32.5% and balanced accuracy of 33.2%, compared with 37.3–40.0% and 35.7–45.0%, respectively, across the four raw-signal models, whose pairwise differences were small relative to their seed-to-seed variability and which are descriptively best interpreted as a single performance tier (no formal test of the within-tier contrasts was performed; see Section 4.3). This gap was directionally consistent across all image-encoding-versus-raw-signal comparisons, though modest rather than overwhelming in effect size for the closest pairs (e.g., the proposed model vs. 1D CNN macro-F1 differed by about 1.1 times their combined standard deviation). This contrasts with the substantially higher performance reported under an earlier, subsequently identified as leakage-affected, evaluation protocol, and illustrates how such evaluation artifacts can inflate the apparent advantage of a more complex, representation-heavy architecture over simpler sequential ones—underscoring the importance of rigorous, inter-patient evaluation protocols when comparing model architectures for ECG arrhythmia classification, and echoing similar concerns raised in the broader literature on evaluation rigor in this field.

At the same time, the comparison was not uniformly unfavorable to the proposed model. A controlled polarity-inversion test showed that the interleaved outer-product image encoding and the proposed model’s classifier are mathematically and empirically invariant to a global sign reversal of the input signal (identical encoded features and predictions for a signal and its exact negation). In contrast, the raw-signal models’ predicted classes changed substantially under the same transformation (e.g., 43–55% of a class-balanced validation subset, and 79–93% of real beats tested directly, for the 1D CNN). This property accounts for the record-117 behavior analyzed in Section 4.4, where the sign inconsistency originated in our own beat-wise alignment heuristic rather than in the physiological signal; the robustness demonstrated is therefore robustness to inconsistent upstream sign decisions. On that record, the 1D CNN’s N-class recall was approximately 53% (with substantial additional instability for LSTM and GRU, 67–84% with seed-to-seed swings of up to 31 percentage points), while the proposed model retained an N-class recall of 98.7% ± 1.0% consistently across all four seeds—the most stable result among all nine models evaluated on this record. The same invariance may also underlie the reduced sensitivity to S and F beats, though this was not tested directly. We consider this a specific, mechanistically grounded contribution of the proposed model, distinct from—and more defensible than—a general classification-accuracy advantage.

The current evaluation relies on synthetically superimposed noise (NST-DB segments added to clean MIT-BIH recordings at controlled SNR levels) rather than naturally occurring recording artifacts, and is intended as a methodological validation under controlled conditions; further studies using naturally noisy recordings and a wider range of datasets are needed to assess broader applicability. The current framework also relies on expert beat annotations for R-peak-centered segmentation, which may not be available for newly collected ECG signals without substituting an automated R-peak detector [26] of uncharacterized sensitivity. In addition, the 192-sample classification window carries little explicit inter-beat timing information; because supraventricular ectopic (S) beats are known to be discriminated substantially by RR-interval irregularity, the near-zero S-class recall observed across all evaluated models may partly reflect this scope limitation; thus, explicit fusion of RR-interval features is a natural direction for follow-up work. In future work, we will repeat the sorting-and-flipping correction ablation with a resolution-matched control and multiple seeds, perform the still-missing downstream denoising ablation under a fixed classifier, evaluate naturally noisy and external datasets (e.g., INCART, PTB-XL, or the MIT-BIH Supraventricular Arrhythmia Database), and explore hybrid architectures combining raw-signal sequential modeling with the proposed model’s polarity-invariant representation.

## Figures and Tables

**Table 1:** Beat types categorized according to the AAMI EC57.

Type	Annotations
Normal (N)	Normal, L/R bundle branch block, Atrial escape, Nodal escape
Supraventricular ectopic beat (S)	Atrial premature, Aberrant atrial premature, Nodal premature, Supraventricular premature
Ventricular ectopic beat (V)	Premature ventricular contraction, Ventricular escape
Fusion beat (F)	Fusion of ventricular and normal
Unknown beat (Q)	Paced, Fusion of paced and normal, Unclassifiable

**Table 2:** Beat-class distribution by partition.

Split	Records	N	S	V	F	Q	Total
Train	33	64,777	2047	5115	423	3899	76,261
Validation	7	12,515	447	1108	364	2080	16,514
Test	8	13,339	287	1013	16	2064	16,719
Total	48	90,631	2781	7236	803	8043	109,494

**Table 3:** Overall denoising performance of the final morphology-aware autoencoder (record-level summary).

Partition	SNR Improvement (dB)	RMSE	PRD (%)	Correlation
Validation	6.71	0.205	40.24	0.870
Test	5.63	0.143	36.89	0.801

**Table 4:** Morphology-preservation evaluation. “Noisy” is the input signal and “denoised” is the autoencoder output; values are means across records.

**(a) Overall**					
**Metric**		**Validation**		**Test**	
		**Noisy**	**Denoised**	**Noisy**	**Denoised**
R-peak amplitude error		1.142	0.430	0.720	0.390
R-peak redetection sensitivity		0.830	0.887	0.812	0.834
R-peak redetection PPV		0.450	0.628	0.372	0.547
RR-interval error (ms)		18.66	10.84	20.23	17.98
T-wave correlation		0.743	0.828	0.710	0.798
P-wave correlation		0.586	0.487	0.647	0.535
QRS-width error (ms)		39.00	14.92	62.63	80.65
**(b) By noise type × SNR level (test partition).**					
**SNR Level**	**Noise Type**	**Metric**		**Noisy**	**Denoised**
−12 dB	BW	R-peak amplitude error		1.3474	0.4315
−12 dB	BW	R-peak redetection sensitivity		0.7688	0.7974
−12 dB	BW	R-peak redetection PPV		0.4198	0.5453
−12 dB	EM	R-peak amplitude error		1.6595	0.5877
−12 dB	EM	R-peak redetection sensitivity		0.5555	0.7207
−12 dB	EM	R-peak redetection PPV		0.2341	0.4334
−12 dB	MA	R-peak amplitude error		1.7787	0.5505
−12 dB	MA	R-peak redetection sensitivity		0.6056	0.7118
−12 dB	MA	R-peak redetection PPV		0.2159	0.4482
12 dB	BW	R-peak amplitude error		0.1149	0.3122
12 dB	BW	P-wave correlation		0.9854	0.6461
12 dB	EM	R-peak amplitude error		0.1268	0.3193
12 dB	EM	P-wave correlation		0.8855	0.6182
12 dB	MA	R-peak amplitude error		0.1178	0.3173
12 dB	MA	P-wave correlation		0.9027	0.6386

(−6/6 dB conditions and QRS-width breakdown omitted from this condensed table).

**Table 5:** Baseline comparison under a frozen, leakage-free evaluation protocol (test-set, clean condition; mean ± std across 4 seeds; best value per metric column in bold).

Model	Input Representation	Accuracy	Macro-F1	Balanced Accuracy	Record-Level Macro-F1
3D CNN baseline	Image encoding	**81.09 ± 0.50**	27.94 ± 1.42	27.28 ± 1.29	20.00 ± 0.41
ResNet-18	Image encoding	78.77 ± 1.03	29.82 ± 0.46	30.10 ± 1.36	20.67 ± 0.35
GoogLeNet	Image encoding	74.24 ± 4.37	27.72 ± 1.67	28.36 ± 2.94	19.84 ± 0.98
DenseNet-121	Image encoding	77.93 ± 3.54	29.64 ± 2.04	30.41 ± 1.16	20.73 ± 0.71
**Proposed**	Image encoding	77.20 ± 1.77	32.52 ± 2.89	33.18 ± 3.33	21.05 ± 0.62
1D CNN	Raw denoised signal	76.24 ± 2.97	39.58 ± 5.55	**44.95 ± 4.97**	22.97 ± 0.83
LSTM	Raw denoised signal	80.78 ± 4.77	37.32 ± 6.60	35.68 ± 4.52	21.97 ± 1.15
GRU	Raw denoised signal	77.76 ± 4.16	38.11 ± 5.17	41.12 ± 3.64	22.73 ± 0.93
CRNN	Raw denoised signal	79.40 ± 3.67	**39.96 ± 3.76**	42.65 ± 2.24	**23.51 ± 0.39**

**Table 6:** Record-Level paired comparison of the proposed model vs. each comparator (macro-F1; 10,000-replicate hierarchical paired-bootstrap 95% CI; exact paired permutation over all 256 record-level sign assignments; Holm correction across 8 comparisons). Results aggregate the 12 noise-type × SNR conditions.

Comparator	Proposed − Comparator (pp)	95% CI	Holm-Corrected *p*
3D CNN baseline	+0.553	−1.740 to 3.071	1.000
ResNet-18	+0.124	−1.316 to 1.676	1.000
GoogLeNet	+0.737	−0.737 to 2.784	1.000
DenseNet-121	+0.172	−1.335 to 1.739	1.000
1D CNN	−2.005	−5.364 to 1.292	1.000
LSTM	−0.954	−2.816 to 0.555	1.000
GRU	−1.593	−4.641 to 1.176	1.000
CRNN	−1.813	−4.355 to 0.531	1.000

**Table 7:** Per-class recall on the test set (clean condition; mean ± std across 4 seeds).

Class	3D CNN	ResNet-18	GoogLeNet	DenseNet-121	Proposed	1D CNN	LSTM	GRU	CRNN
N	98.44 ± 0.55	94.01 ± 1.49	88.69 ± 6.27	92.54 ± 5.24	89.31 ± 2.71	82.48 ± 1.52	91.58 ± 3.36	85.94 ± 3.52	87.50 ± 2.97
S	0.00 ± 0.00	0.17 ± 0.20	2.00 ± 3.78	0.00 ± 0.00	0.00 ± 0.00	18.99 ± 10.80	0.09 ± 0.17	7.84 ± 7.91	9.84 ± 7.81
V	33.86 ± 4.49	50.67 ± 8.88	45.58 ± 15.57	51.65 ± 7.42	55.80 ± 15.04	81.29 ± 2.68	47.63 ± 12.88	75.44 ± 6.35	74.75 ± 8.04
F	0.00 ± 0.00	0.00 ± 0.00	0.00 ± 0.00	0.00 ± 0.00	0.00 ± 0.00	0.00 ± 0.00	0.00 ± 0.00	0.00 ± 0.00	1.56 ± 3.13
Q	4.11 ± 2.87	5.66 ± 2.49	5.50 ± 0.94	7.87 ± 3.76	20.81 ± 16.07	41.98 ± 25.79	39.12 ± 26.07	36.37 ± 16.13	39.61 ± 11.73

**Table 8:** N-class specificity of the proposed model by SNR level, illustrating the source of its macro-specificity gap (test set; mean across 4 seeds and the three noise types, all four SNR levels).

SNR Level	N-Class Specificity (%)	S/V/F/Q-Class Specificity, Range (%)
−12 dB	26.04	94.59–99.90
−6 dB	28.22	95.25–99.94
6 dB	32.06	95.23–99.94
12 dB	34.28	95.19–99.92

**Table 9:** Classification performance by noise type (macro-F1, %; mean across 4 seeds and the 4 SNR levels within each noise type). All noise aggregates BW, EM, and MA across all four SNR levels.

Model	BW	EM	MA	All Noise
3D CNN	26.95	25.40	25.83	26.06
ResNet-18	28.37	26.47	27.28	27.38
GoogLeNet	26.77	25.32	25.97	26.02
DenseNet-121	28.10	26.30	26.86	27.09
Proposed	30.09	28.43	29.40	29.31
1D CNN	37.85	34.55	37.00	36.47
LSTM	35.71	32.72	34.59	34.34
GRU	36.17	33.25	35.29	34.90
CRNN	37.33	33.82	35.99	35.72

**Table 10:** Classification performance by SNR level, aggregated across noise types (macro-F1, mean ± std across 4 seeds and three noise types).

Model	−12 dB	−6 dB	6 dB	12 dB
3D CNN baseline	23.30 ± 1.77	25.25 ± 1.61	27.68 ± 1.22	28.01 ± 1.39
ResNet-18	24.41 ± 2.00	26.53 ± 1.50	29.02 ± 0.65	29.52 ± 0.47
GoogLeNet	23.74 ± 1.87	25.57 ± 1.66	27.27 ± 1.12	27.51 ± 1.29
DenseNet-121	24.18 ± 2.05	26.22 ± 1.67	28.54 ± 1.61	29.40 ± 1.77
Proposed	25.72 ± 2.66	28.33 ± 2.03	31.16 ± 2.27	32.04 ± 2.60
1D CNN	31.55 ± 4.54	35.48 ± 4.18	39.19 ± 4.76	39.64 ± 4.90
LSTM	29.97 ± 3.73	33.27 ± 3.55	36.81 ± 5.37	37.30 ± 5.86
GRU	30.07 ± 3.70	34.17 ± 3.15	37.39 ± 3.97	37.97 ± 4.28
CRNN	30.27 ± 4.18	34.27 ± 3.03	38.66 ± 3.16	39.66 ± 3.36

**Table 11:** Correction ablation: effect of sorting and flipping within the image-encoding pipeline (single seed; interpret with appropriate caution).

Encoding Variant	Record Macro-F1			Test Clean (Pooled Macro-F1)
	Validation Clean	Test Clean	Test All-Noise	
Outer product only (no correction)	0.3115	0.1984	0.1900	0.3481
+ Sorting	0.2679	0.2141	0.2039	0.3409
+ Sorting + Flipping (proposed)	0.3018	0.2029	0.1942	0.3523

**Table 12:** Model efficiency: inference time and parameter count.

Model	Parameters	Test Clean (ms/Sample)
Proposed	15,554,085	~4.2–4.4
1D CNN	779,397	~4.0–4.2

## Data Availability

The MIT-BIH Arrhythmia Database and the Noise Stress Test Database analyzed in this study are publicly available through PhysioNet [22,23].
